# Identifying victimisation profiles in people with psychosis and a history of childhood trauma: a latent class analysis

**DOI:** 10.1080/17522439.2021.2009903

**Published:** 2021-12-21

**Authors:** Georgina L Barnes, Richard Emsley, Philippa Garety, Amy Hardy

**Keywords:** Psychosis, childhood trauma, trauma-informed care, routine enquiry, latent class analysis

## Abstract

**Background:**

People with psychosis experience higher rates of childhood victimisation compared to the general population, which may impact on mental health and recovery. This study aimed to identify childhood victimisation profiles in a clinical sample to inform recommendations for routine care.

**Methods:**

Participants were 146 adults (ages 19–65 years; M = 42.2) with schizophrenia-spectrum diagnoses reporting trauma. Childhood trauma was assessed using two retrospective measures, and a latent class analysis (LCA) was performed on four trauma types (sexual abuse, emotional abuse, physical abuse and neglect). Multinomial logistic regression investigated demographic differences between the classes.

**Results:**

Four distinct childhood trauma classes were identified: Emotional abuse/neglect (n = 29); physical abuse (n = 14); sexual abuse (n = 19); and poly-victimisation (n = 84). There were no differences between the classes in terms of age, ethnicity, relationship status, education or current employment (relative risk (RR) = 0.85–1.27, *p* > 0.05). Participants in the poly-victimisation class were significantly more likely to be female (RR = 0.22–0.28, *p* < 0.04).

**Discussion:**

Adults with psychosis, particularly females, are likely to report poly-victimisation in childhood. This highlights the need to comprehensively but concisely assess experiences of abuse and neglect in clinical care, in line with trauma-informed approaches.

## Introduction

People with psychosis report higher rates of childhood interpersonal trauma compared to the general population, with evidence that this can detrimentally impact mental health and recovery (see, Alameda et al., 2021; Bloomfield et al., 2021). To address trauma-related needs in people with psychosis, Trauma Informed Care (TIC) is recommended as an organisational approach for the delivery of mental health care in the UK (Sweeney et al., 2018). Trauma-informed organisations *realise* the widespread impact of trauma for society, *recognise* trauma (including routine enquiry), *respond* by providing trauma-focused interventions when needed, and *resist* re-traumatisation for people accessing mental health services (Sweeney et al., 2019).

In the UK National Health Service (NHS), routine enquiry about violence and abuse has been recommended since 2003, and in 2008 was enshrined in policy for all people under the Care Programme Approach (Department of Health, 2008; Scott & McNeish, 2008). The ‘Responding Effectively to Violence and Abuse” (REVA) policy in the UK specifies that all clinical staff should be trained to implement the “abuse question” during mental health assessments (i.e. “*Have you experienced physical, sexual or emotional abuse at any time in your life?”)*. However, a recent audit of the REVA policy found that only 17% of service users from 53 NHS Trusts were recorded as being asked about experiences of violence and abuse (Brooker et al., 2016), despite an earlier initiative that aimed to improve REVA implementation (Scott et al., 2015). An obstacle to implementing the abuse question into routine care may be that the REVA policy is not in line with best practice recommendations for trauma assessment, including the use of prompts that enquire about specific behaviours or events (Read et al., 2020).

In addition to the poor implementation of routine enquiry, a further concern is that the broad “abuse” question may not sufficiently recognise an individual’s trauma history or experiences that are subjectively perceived as threatening; such as childhood neglect, other attachment-disrupting events (e.g. parental loss), psychosis-related traumas and adverse treatment experiences, all of which may impact upon mental health and contact with services. This issue also applies to common measurement tools. For example, the Adverse Childhood Experiences (ACE) checklist only focuses on recurrent experiences of abuse and neglect (i.e. those occurring “often” or “very often”) within the home from caregivers and misses other victimisation contexts, such experiences of racism, peer bullying, or assaults occurring outside of the home, as well as mental health-related traumas (Lacey & Minnis, 2020). This is important to consider in psychosis, given the body of evidence that shows that people with psychosis commonly report co-occurring trauma types across a range of contexts (Shevlin et al., 2013; Van Dam et al., 2015).

Examining the occurrence of childhood victimisation types and contexts in psychosis will be useful for improving policy and practice in relation to routine enquiry, as better understanding of trauma profiles in this population will help to guide how we should ask about traumatic events in routine care. Latent class analysis (LCA) may be an alternative and helpful approach to capture different typologies of victimisation in people with psychosis. LCA is a clustering technique that groups individuals into “classes” based on the trauma types they report. The focus of this method is to examine how prevalent different combinations of victimisation experiences are, thereby overcoming the limitation of assessing individual trauma types in isolation (Roesch et al., 2010). The LCA method has recently been applied to the field of traumatology. A recent systematic review of 17 studies (O’Donnell et al., 2017) found evidence for homogenous patterns of lifetime trauma exposure for those reporting victimisation, characterized by sexual trauma, non-sexual trauma (i.e. physical abuse, emotional abuse and/or neglect) and poly-victimisation. There was also some evidence that risk of psychiatric diagnoses was highest for poly-victimisation classes. LCA may therefore be a useful method in traumatology research, as it identifies prevalence combinations of victimisation experiences, rather than relying on cumulative trauma scores (Lacey & Minnis, 2020). The LCA approach has been used to identify patterns of victimisation in the general population and relate this to psychosis diagnosis (Boyda et al., 2020; Houston et al., 2011; Murphy et al., 2014; Shevlin et al., 2013). To date, only a limited number of studies have used LCA to identify discrete patterns/profiles of childhood victimisation in a clinical psychosis sample (Albers et al., 2018; Lau et al., 2021). The aim of this study was therefore to identify latent classes of childhood victimisation in adults with psychosis and a history of trauma to inform recommendations for trauma assessment in clinical practice.

## Materials and methods

### Participants

The sample consisted of participants recruited to two linked randomized experimental studies investigating cognitive mechanisms in delusions (Freeman et al., 2014; Garety et al., 2015: ISRCTN06118265). Participants aged 18–65 years with a schizophrenia-spectrum diagnosis (Non-affective, ICD-10 F20-F28) and a current delusion, assessed on the Schedules for Clinical Assessment in Neuropsychiatry (SCAN; Wing et al., 1990), were recruited from community psychosis teams across six mental health services in the UK. Exclusion criteria were primary diagnoses of substance misuse, organic syndrome or intellectual disability, or insufficient English to complete the research assessment measures. As our focus was to identify patterns of childhood victimisation in those with a history of trauma, an additional exclusion criterion for the current study was not reporting any childhood trauma on the research measures.

### Design and procedure

Demographic data (age, sex, ethnicity, relationship status, years of education and current employment status) were collected at baseline and trauma measures were collected at a three-month follow-up. The current study was a cross-sectional design. Trauma Assessment Measures

The Trauma History Questionnaire (THQ; Green, 1996) is a structured interview that assesses lifetime exposure to victimisation and non-victimisation trauma. This includes discrete, episodic or persistent events. All events were coded according to childhood, adulthood and lifetime incidence. Trauma type was coded into non-victimisation (i.e. accident, illness, natural disasters) and victimisation (i.e. sexual, physical and emotional abuse). Childhood Sexual Abuse (CSA) was coded from the presence of at least one of the two items assessing unwanted sexual contact and rape prior to 16 years. Childhood Physical Abuse (CPA) was coded from the presence of at least one of the two items assessing physical assault (with and without a weapon) and a bullying item that was reviewed for reports of interpersonal violence. Childhood Emotional Abuse (CEA) was coded from the presence of a bullying item that was reviewed for reference to psychological victimisation. CEA was coded if physical harm or injury were not endorsed on the item. Lastly, event descriptions for the remaining items were reviewed for reference to victimisation and coded into CSA, CPA or CEA. The THQ has acceptable psychometric properties for use in psychosis samples (Hooper et al., 2011).

The Childhood Experience of Care and Abuse Questionnaire (CECA-Q; Smith et al., 2002) is a self-report measure assessing caregiving experiences from mother and father figures before the age of 17 years. The Antipathy and Neglect subscales are made up of 16 items (eight for each caregiver) rated on a 5-point scale, with higher scores reflecting greater severity. Childhood Antipathy and Childhood Neglect (CN) were coded as present or absent based on standardised severity cut-offs (maternal/paternal antipathy ≥ 25; maternal neglect ≥ 22; paternal neglect ≥ 24). The CECA-Q has well-established validity and reliability (Bifulco et al., 2005).

### Data analysis

All analyses were carried out using Stata version 15.0 (StataCorp, 2017).

### Demographic variables

A range of demographic variables were used in the analysis. Age and education (years) were entered as continuous variables. Sex was coded into a dichotomous variable, which identified participants as male (0) or female (1). For the purpose of the multivariate analyses, ethnicity was re-coded into a dichotomous variable, which identified respondents as being from a white ethnic background (0) or a black or minority ethnic background (BME) (1). Participants were asked if they were in employment, education or training at the time of interview and this was re-coded into a dichotomous variable that identified respondents as unemployed (0) or employed (1). Lastly, participants were asked about their relationship status and this was coded into a dichotomous variable that identified respondents as single/separated (0) or married/in a relationship (1).

### Childhood trauma

Responses to individual trauma items were coded as binary variables (1 = Yes; 0 = No) and a separate variable was then created for each childhood trauma category indicating if one or more questions in each was endorsed. This resulted in four binary variables representing the four categories of childhood victimisation: CSA (THQ); CPA (THQ); CEA (THQ Bullying and CECA-Q Parental Antipathy combined) and CN (CECA Parental Neglect).

Latent Class Analyses (LCA) were performed on the four categorical trauma variables Two to six-class solutions were considered based on recommendations for applying LCA in trauma research (Lanza & Cooper, 2016). The models were estimated using robust maximum likelihood. To avoid solutions based on local maxima, 10 random sets of starting values were used initially and 100 final-stage optimisations. Three theory-based model fit indices were used to decide the best-fitting solution for the data: the maximum log-likelihood, the Akaike Information Criterion (AIC) (Akaike, 1987) and Bayesian Information Criterion (BIC; Schwarz, 1978). The model that produces the lowest values demonstrates better fit. Entropy values were also used as a measure of classification uncertainty, with values >0.8 indicating good separation between the classes (Celeux & Soromenho, 1996).

Interpretation of the class solutions was based upon the percentage of the sample estimated to belong to a class (class membership probabilities) and the percentage of participants in each class estimated to have been exposed to each trauma indicator (item response probabilities). Class solutions that included small class sizes below 5% of the sample (<9 participants) were deemed to have poor predictive value and were not accepted.

Last, multinomial logistic regression was employed to determine associations between the latent trauma classes and demographic variables. The dependent variable was trauma class and the predictors were the six demographic variables. Associations were evaluated using relative risk probabilities and 95% confidence internals (CIs) through the mlogit function. Relative risk ratios (RR) were reported as a measure of effect size.

## Results

### Rates of childhood victimisation in the sample

In total, 146 participants completed both trauma measures and reported at least one victimisation trauma and were included in the analysis. Missing scores for the four variables ranged from 2.3% to 6.4% across the sample. The most commonly endorsed victimisation experiences were emotional bullying (50.3%), unwanted sexual experiences (35.5%) and physical abuse at home (34.7%). The least common were forced sexual intercourse (16.8%), assault with a weapon (18.0%) and maternal neglect (19.2%). Table 1 shows the frequencies of childhood victimisation exposure reported in the sample.

**Table 1.** Rates of childhood victimisation types (n = 146).

Victimisation type	N	%
Sexual abuse^a^	64	43.8%
Unwanted sexual experience	59	40.4%
Forced sexual intercourse	28	19.2%
Physical abuse^a^	93	63.7%
Assault with weapon	30	20.5%
Assault without weapon	35	24.0%
Physical abuse at home	58	39.7%
Bullying (physical)	34	23.3%
Emotional abuse	105	71.9%
Bullying (emotional)	84	57.5%
Mother antipathy	53	36.3%
Father antipathy	55	37.7%
Neglect^b^	67	45.9%
Mother neglect	32	21.9%
Father neglect	48	32.9%

^a^n = 141, ^b^ n = 145

### Latent class models of trauma exposure

LCA was performed on available data from the 146 participants reporting at least one type of childhood trauma using Maximum Likelihood estimation. Table 2 outlines the fit indices for the sequential class solutions, with the best fitting model statistics highlighted in bold. AIC values decreased up to the 5-class solution, indicating that the 5-class solution was a poorer fit than the 4-class solution. The 6-class solution identified a latent class comprised only 5 (3%) participants, which was considered to have inadequate predictive utility. The 4-class solution had the lowest AIC and BIC values of all the solutions, demonstrating a better fitting model. Entropy values were <0.8 for all class solutions (0.417–0.640) demonstrating relatively poor separation of the latent classes. However, entropy was highest for the 4-class solution and this was therefore accepted.

**Table 2.** Fit information for the five latent class solutions (n = 146).

Classes	df	LL^a^	AIC	BIC	Entropy
2	9	−354.08	726.17	752.64	0.640
3	13	−348.18	722.36	760.60	0.626
4	14	−340.56	709.13	750.31	0.640
5	22	−336.51	717.02	781.74	0.550
6	22	−335.58	715.15	779.87	0.417

^a^Loglikelihood for class solution

### Description of the classes

**Figure 1.**
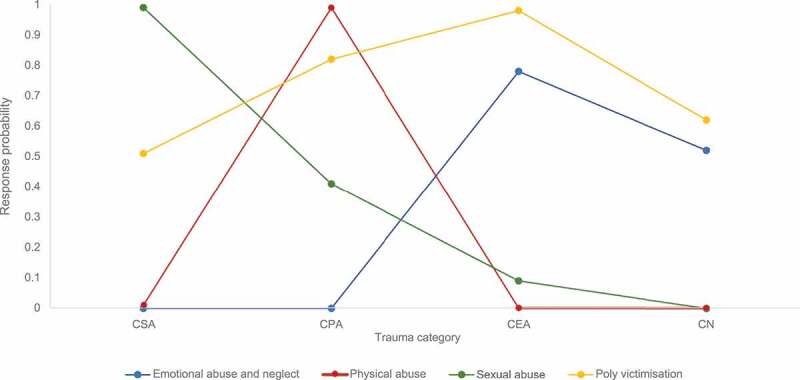
Latent class profile plot for the 4-class model (n = 146).

The profile plot for the final 4-class solution is shown in Figure 1. Class 1 (16%) was characterised by high response probabilities for CEA (78%) and CN (52%) relative to other abuse types (0%). This was labelled the “emotional abuse and neglect class”. Class 2 (10%) had high response probabilities for CPA (99%) relative to the other abuse types (0–1%). This was labelled the “physical abuse” class. Class 3 (14%) had high response probabilities for CSA (99%) relative to other abuse types (0–41%). This class was labelled the “sexual abuse” class. Lastly, Class 4 (60%) was characterised by high to moderate response probabilities for all child trauma types (51–98%). This class was labelled the “poly-victimisation” class. Table 3 outlines the class membership and item response probabilities for each trauma indicator across the four classes.

**Table 3.** Latent class probabilities for the 4-class model (n = 146).

	Class 1: Emotional abuse & neglect (n = 29)	Class 2: Physical abuse (n = 14)	Class 3: Sexual abuse (n = 19)	Class 4: Poly victimisation (n = 84)
Pr (Class)	0.16	0.10	0.14	0.60
*Probability of*				
Child sexual	0.00	0.01	0.99	0.51
Child physical	0.00	0.99	0.41	0.82
Child emotional	0.78	0.00	0.09	0.98
Child neglect	0.52	0.00	0.00	0.62

### Demographic characteristics of the classes

Table 4 displays the demographic characteristics of the sample by trauma class membership. Class differences for the demographic variables were examined using multinomial logistic regression. Class 4 was used as the base outcome to compare those exposed to poly-victimisation relative to the other three trauma classes.

**Table 4.** Demographic characteristics of the sample by class (n = 146).

	Emotional abuse/ neglect (n = 29)	Physical abuse (n = 14)	Sexual abuse (n = 19)	Poly victimisation (n = 84)
Age, M (SD)				
	42.7 (10.7)	38.6 (8.4)	45.1 (12.9)	42.1 (10.7)
Sex, N (%)				
*Male*	22 (75.9)	11 (78.6)	13 (68.4)	40 (47.6)
*Female*	7 (24.1)	3 (21.4)	6 (31.6)	44 (52.4)
Ethnicity, N (%)				
*White British/ Other*	21 (72.4)	6 (42.9)	10 (52.6)	48 (57.1)
*Black British/ Caribbean/African*	4 (13.8)	8 (57.1)	4 (21.1)	26 (31.0)
*Asian British/Other*	2 (6.9)	0 (0.0)	1 (5.3)	2 (2.4)
*Mixed Heritage*	2 (6.9)	0 (0.0)	3 (15.8)	7 (8.3)
*Other*	0 (0.0)	0 (0.0)	1 (5.3)	1 (1.2)
*Relationship status, N (%)*				
*Single/separated*	24	14	15	77
*Married/In a relationship*	5	0	4	6
*Education (years) M (SD)*				
	12.1 (3.4)	11.2 (2.7)	11.8 (3.6)	10.5 (2.9)
*Employed or in training*				
*Yes*	2 (6.9)	3 (21.4)	3 (15.8)	4 (4.8)
*No*	27 (93.1)	11 (78.6)	16 (84.2)	80 (95.2)

Table 5 outlines the results of the multinomial regression. Participants in the poly-victimisation class were significantly more likely to identify as female compared to those in the emotional abuse/neglect class (RR = 0.22; CI = 0.08–0.62, *p* = 0.005) and the sexual abuse class (RR = 0.28; CI = 0.08–0.95, *p* = 0.04). There was evidence that participants in the poly-victimisation class (relative to the emotional abuse/neglect class) were more likely to identify as being from a black or minority ethnic background, however this did not reach statistical significance (RR = 0.38; CI = 0.14–1.04, p = 0.06). There were no other significant differences between the classes in terms of age, relationship status, education level or current employment (RR values all = 0.85–1.27, *p* values >0.05)

**Table 5.** Multinomial logistic regression of associations between the latent classes and the demographic variables (n = 146).

	Emotional/neglect abuse (class 1)		Physical abuse (class 2)		Sexual abuse (class 3)	
	RR	CI	RR	CI	RR	CI
Age						
	1.01	0.97–1.06	0.99	0.93–1.04	1.03	0.98–1.09
Sex						
*Female*	0.22**	0.08–0.62	0.27	0.06–1.09	0.28*	0.08–0.95
Ethnicity						
*BME*	0.38	0.14–1.04	1.57	0.47–5.23	1.06	0.36–3.12
Relationship status						
*Married/in a relationship*	1.27	0.71–1.54	1.61	0.63–1.98	4.18	0.96–4.78
Education (years)						
	1.06	0.90–1.25	1.14	0.93–1.41	1.04	0.85–1.26
Current Employment						
Yes	0.85	0.08–8.75	5.04	0.88–8.77	4.71	0.82–6.87
Log likelihood = −143.52						

Base outcome = Class 4 (Poly-victimisation); Model χ^2^ = 33.16; df = 3; p = 0.01, n = 146; *p < 0.05, **p < 0.01; RR = relative risk ratio

## Discussion

This study is, to our knowledge, the first to identify latent classes of victimisation in adults with psychosis-spectrum conditions reporting childhood interpersonal trauma. Using this approach, we identified four clear childhood victimisation classes, characterised by participants reporting emotional abuse and neglect (20%), physical abuse (10%), sexual abuse (13%) and poly-victimisation (57%). These results build on a previous review that identified discrete patterns of trauma exposure in clinical and non-clinical samples (O’Donnell et al., 2017) and indicate that similar childhood victimisation patterns may be found in people with schizophrenia-spectrum diagnoses.

The prevalence of poly-victimisation in our sample was higher than the rates reported in the O’Donnell et al. review, which ranged between 1.3% and 9% for general population samples, and 37–50.5% for clinical samples (though this only included samples of fostered youth and females with substance use difficulties). Our findings show that poly-victimisation is highly prevalent in people with psychosis accessing clinical services, and this is consistent with previous research (e.g. Albers et al., 2018; Shevlin et al., 2013). We also found that female participants were significantly more likely to have been exposed to all childhood abuse and neglect types and contexts compared to males, which is in line with existing LCA studies using non-clinical samples (Haahr-Pedersen et al., 2020). This also links to research that shows gender variation in rates of poly-victimisation in people with psychosis (Fisher et al., 2009; Pruessner et al., 2019).

These findings should be placed in the context of previous research, which shows that traumatic events are also common in the general population. For example, in a large epidemiological study across 24 countries, 70.4% of people reported lifetime traumas (Kessler et al., 2017). However, rates varied significantly across the 20 trauma types assessed, with non-victimisation events more prevalent than victimisation traumas. For example, death of a loved one/witnessing a trauma experienced by another person accounted for over 40% of all trauma exposures, 25% were accidents, whereas sexual violence and physical violence were reported by 10% and 14% of people respectively. However, despite trauma being widely prevalent in the general population, evidence suggests particularly high rates of interpersonal trauma and poly-victimisation in psychosis (De Vries et al., 2018), both childhood and adulthood, which appears consistent across a range of assessment methods and populations (Hardy et al., 2020). Evidence also shows higher rates of poly-victimisation in other clinical groups, e.g. major depression and bipolar disorder (Albers et al., 2018). Our results, taken in the context of this large body of research, therefore support calls for comprehensive but concise routine assessment measures in clinical services.

This study has important implications for implementing trauma-informed services for people with psychosis (Bush & Brennan, 2018; Mitchell et al., 2020) and the need to comprehensively but concisely assess trauma in this population. The findings suggest that guidelines which take a broad approach to routine trauma enquiry, and those that focus on assessing specific abuse types or contexts, may not sufficiently recognise different patterns of childhood victimisation, which is important given that adults with psychosis are likely to report co-occurring childhood trauma experiences both inside and outside of the home. Such lines of enquiry may also miss trauma events and contexts that are subjectively perceived as threatening, such as childhood neglect and other attachment disrupting events. Practitioners therefore require training and support to realise and recognise trauma in people accessing clinical services. This should focus on increasing awareness of the prevalence and consequences of poly-victimisation for people with psychosis and recognising trauma through comprehensive routine enquiry that asks about different types of abuse and neglect using behaviourally specific prompts (Read et al., 2020). Further research may now focus on how different victimisation profiles relate to patterns of help-seeking, service engagement and therapeutic relationships in psychosis, which could further guide trauma-informed support (e.g. Bucci et al., 2015).

Limitations of the study should be considered. First, sample bias may have occurred given the experimental studies from which the sample was taken (Freeman et al., 2014; Garety et al., 2015) who were adults recruited for high conviction delusional beliefs in the context of schizophrenia-spectrum diagnoses. It will be helpful to use latent variable modelling approaches, such as LCA, in other clinical samples (e.g. those with an identified at-risk mental state or first-episode psychosis) to see if similar childhood victimisation patterns emerge which may help to build on the current evidence base (O’Donnell et al., 2017).

Second, although we assessed a broad range of childhood traumas, we did not comprehensively assess all events that may be relevant to people with psychosis. For example, racism and discrimination, peer bullying, attachment-disrupting events (e.g. parental loss), psychosis-related traumas (e.g. voices or visions that are distressing) and adverse treatment experiences (e.g. involuntary admissions, use of physical restraints) are also common in people with psychosis and may impact on recovery (Alameda et al., 2021; Bloomfield et al., 2021). We recommend that future studies and routine practice incorporate a broader range of trauma measures. For example, the Trauma and Life Events (TALE) checklist (Carr et al., 2018) is the first routine screening tool designed for trauma assessment in psychosis services and may address the limitations around current routine enquiry policy in clinical practice.

Another limitation of the study is that we collapsed individual trauma items into four categories and did not analyse them individually. We decided these four categories a priori to identify whether current recommendations around routine trauma enquiry are sufficient, or whether they may risk missing common trauma profiles in psychosis (such as poly-victimisation, including neglect). However, we recognise this may have obscured specific trauma profiles, and future research investigating victimisation profiles in psychosis could include a broader range of trauma types. Lastly, we only included retrospective trauma measures that may be subject to biases in remembering and disclosure (Baldwin et al., 2019). Where possible, future studies should seek to combine prospective alongside retrospective assessment to more rigorously understand lifetime victimisation patterns in people with psychosis.

To conclude, this study supports the need for mental health services to address trauma-related needs in people with psychosis. Comprehensive routine enquiry and assessment of lifetime abuse and neglect will facilitate better trauma-informed care planning, including active risk management and safeguarding interventions for those at risk of (re)victimisation. Organisations should also aim to promote safe and effective responding for those who do disclose trauma, by providing access to trauma-focused interventions when indicated. This is a timely endeavour given calls for TIC to be implemented in psychosis services (Mitchell et al., 2020; NHS England, 2019) and emerging evidence that trauma therapies support recovery and reduce rates of re-victimisation in people with psychosis (Brand et al., 2019; Van Den Berg et al., 2020).
